# Focal Segmental Glomerulosclerosis in Waldenström's Macroglobulinemia

**DOI:** 10.1155/2020/8895705

**Published:** 2020-08-27

**Authors:** Akshee Batra, Shatha Herz Allah, Bijin Thajudeen

**Affiliations:** Department of Nephrology, Banner University of Arizona Medical Center, 1501 North Campbell Ave, Tucson, AZ, USA

## Abstract

Renal involvement in Waldenström's macroglobulinemia is a rare manifestation. Although most renal involvement is due to monoclonal immune deposits, pathology can also be unrelated to it. Here, we describe a 68-year-old female with a history of Waldenström's macroglobulinemia who presented with generalized edema and nephrotic range proteinuria. A renal biopsy showed findings consistent with focal segmental glomerulosclerosis. Treatment with oral prednisone leads to the resolution of proteinuria. This case highlights the importance of identifying pathology that might not be directly related to monoclonal gammopathy, which could have implications on the management.

## 1. Introduction

Waldenström's macroglobulinemia (WM) is a lymphoproliferative disorder involving B cells characterized by an IgM monoclonal protein >1 g/dl and 10% lymphoplasmacytic infiltrate in the bone marrow [1]. Kidney disease is a common presentation of WM usually characterized by IgM deposits along the glomerular basement membrane (GBM), infiltration of the interstitium, or amyloidosis [1, 2]. However, occasionally the pathology could be unrelated to the immunoglobulin deposition (Tables 1 and 2). Here, we report a case of focal segmental glomerulosclerosis (FSGS) in a patient with WM. According to our knowledge, there is only one other reported case of FSGS in WM [1].

## 2. Case Report

**Figure 1:**
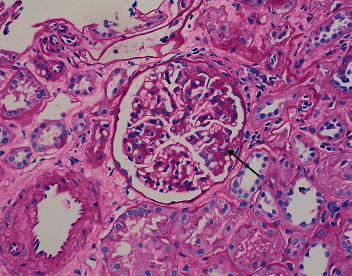
Light microscopy showing glomeruli with segmental sclerosis and attachment of the glomerular tuft to Bowman's capsule (arrow) (H&E stain).

**Figure 2:**
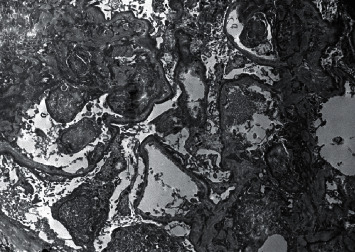
Electron microscopy showing more than 90%-foot process effacement.

A 68-year-old female presented to the outpatient clinic with complaints of lower extremity edema for four weeks. Her past medical history was significant for lymphoplasmacytic lymphoma with IgM monoclonal gammopathy or WM. Since the criteria for treatment were not met, she did not receive any treatment for WM. Her home medications included omeprazole and ferrous sulfate. Her vitals showed a temperature of 98°F, blood pressure of 136/78 mmHg, pulse rate of 84/minute, respiratory rate of 16/min, and body mass index of 22.35 kg/m^2^. The rest of the physical examination was unremarkable except for 2+ edema of the lower extremities. Laboratory studies yielded the following values: hemoglobin 8.3 g/dl, white blood cell count 14400/mm^3^, platelet count 496000/mm^3^, blood urea nitrogen 13 mg/dl, creatinine 0.6 mg/dl, calcium 8.5 gm/dl, erythrocyte sedimentation rate (ESR) 94 mm/1st hour, LDH 340 IU/I, and total protein 7.5 g/dl with albumin 3.9 g/dl. Urine sediments contained few erythrocytes and hyaline casts. The spot protein/creatinine ratio was 16.2 gm/gm creatinine, and the albumin-to-creatinine ratio was 9.9 gm/gm creatinine. Serum immunoelectrophoresis showed IgM 1693 mg/dl, IgG 453 mg/dl, and IgA 23 mg/dl. Serum immunofixation revealed monoclonal IgM kappa light chains. The concentration of serum kappa and lambda light was 73.74 mg/L and 2.38 mg/L, respectively. The ratio of kappa to lambda was 30.98. Peripheral blood flow cytometry revealed a monocytic kappa light-chain-restricted B-cell population. Cryoglobulins were absent. All serologies, including complements, ANA, anti-dsDNA, ANCA, HIV, and hepatitis panel, were negative. A renal biopsy was performed. Light microscopy showed glomeruli with thin and delicate capillary loops, three glomeruli showed segmental sclerosis, and two glomeruli demonstrated mild attachment to Bowman's capsule without significant tubulointerstitial inflammation (Figure 1). Immunofluorescence showed a tubular cast that stained for IgG, IgM, and kappa. Electron microscopy revealed more than 90%-foot process effacement without any evidence of immune deposits (Figure 2). The final diagnosis was primary glomerulosclerosis. Treatment with oral prednisone was started at 60 mg daily, and the dose of prednisone was tapered and stopped over eight months. Additional treatment included losartan for proteinuria and Bactrim as well as fluconazole for infection prophylaxis (stopped after six months). Protein/creatinine ratio improved to 330 mg/gm creatinine at the end of eight months. There was a complete disappearance of edema of extremities. The plan is to continue monitoring without any maintenance treatment.

## 3. Discussion

Pathogenesis of renal involvement in WM can range from direct infiltration of the lymphoplasmacytic cells, glomerular or tubular deposition of paraprotein, or an immune-mediated reaction [1–3]. Glomerular involvement due to paraprotein includes amyloid related and nonamyloid related, of which amyloid light-chain- (AL-) related glomerulopathy is the most common [1–3]. Among the nonamyloid glomerulopathies, cryoglobulinemia is the most reported renal pathology [1, 2]. Non-paraprotein-mediated kidney pathologies have also been reported, including minimal change disease, focal segmental glomerulosclerosis, membranous glomerulopathy, and thrombotic microangiopathy [1, 3].  Table 2 shows a diverse array of kidney diseases reported in WM patients.

This case highlights an unusual presentation of FSGS in kidney biopsy of a patient with prediagnosed WM. FSGS pathogenesis involves a sequel of glomerular podocyte damage and dysfunction [4]. However, at the same time, the pathogenesis of FSGS in WM is not well defined in the literature. The three possible explanations for the presence of FSGS in this patient include idiopathic FSGS unrelated to WM, paraprotein-induced podocyte injury, and paraneoplastic phenomenon affecting the genes associated with cytoskeleton organization and podocyte signaling [1–3, 5]. In the largest published study on IgM-producing B-cell lymphoproliferative disorders, Higgins et al. revealed the presence of one FSGS from the kidney biopsies of 44 patients [1]. In another cohort where 44 patients had pathology related to WM, two of the patients had minimal change, and one had membranous nephropathy [3]. Due to limited literature and failure to establish a direct causal relationship with WM, we ascertained the diagnosis as primary FSGS unrelated to WM.

Treatment for renal manifestations in WM consists of supportive therapy with hydration, plasmapheresis, and combination chemotherapy for underlying hematologic malignancy [6]. Novel therapeutics like bortezomib, lenalidomide, and ibrutinib can be considered [7]. An intervention like autologous stem cell transplantation has also been found to be effective on a case-to-case basis [8].  Table 1 shows the medications used in the treatment of WM. Since there was no histopathological finding relating the pathogenesis to paraprotein, we decided to treat this as a primary FSGS. The presence of immune deposits in immunofluorescence or electron microscopy could have favored the treatment of WM rather than primary FSGS. This case highlights the importance of individualizing therapy according to clinical presentation and kidney biopsy results.

## Figures and Tables

**Table 1:** Clinical studies, types of renal disease, and treatment in Waldenström's macroglobulinemia.

Study	Number of patients	Pathology seen	Treatment
Morel-Maroger et al. [2]	16 patients	3 amyloidosis 6 nonamyloid glomerular disease (3 cryoglobulinemia) 7 had no detectable lesions	Not available

Higgins et al. [1]	44 patients	17 amyloidosis 9 nonamyloid glomerular disease 7 tubulointerstitial nephritis 9 non-monoclonal gammopathy-related glomerular disease (1FSGS)	Amyloid glomerulopathy: Rituximab R Ituximab Chemotherapy Bortezomib Nonamyloid glomerulopathy: Steroid Rituximab Rituxima Bendamustine Bortezomib Chemotherapy

Vos et al. [3]	44 patients	Amyloidosis 11 Cryoglobulinemia 10 Light-chain deposition disease 4 Cast nephropathy 4 TMA 3 Minimal change disease 2 Membranous nephropathy 1 LPL infiltration 8 Light-chain tubulopathy 1	Proteosome inhibitor + rituximab Alkylator + rituximab Nucleoside analogue + rituximab Bendamustine Single-agent rituximab

LPL: lymphoplasmacytic lymphoma infiltration; TMA: thrombotic microangiopathy.

**Table 2:** Renal diseases associated with Waldenström's macroglobulinemia.

Amyloid glomerulopathy	Nonamyloid glomerulopathy	Tubulointerstitial lesions	Non-paraprotein-related lesions
Monoclonal light-chain amyloidosis	Cryoglobulinemic GN	Light-chain cast nephropathy	Minimal change disease
Monoclonal heavy-chain amyloidosis	Non-cryoglobulinemic GN	Lymphoma infiltration	Thrombotic microangiopathy
Monoclonal light- and heavy-chain amyloidosis	Light- and/or heavy-chain deposition disease	Lymphoma infiltration with light-chain cast nephropathy	Membranous GN
	Proliferative glomerular diseases		Acute tubular necrosis
	Immunotactoid glomerulopathy		Diabetic nephropathy
			FSGS

GN: glomerulonephritis; FSGS: focal segmental glomerulosclerosis.
